# Analysis of the Formation of Sauce-Flavored Daqu Using Non-targeted Metabolomics

**DOI:** 10.3389/fmicb.2022.857966

**Published:** 2022-03-24

**Authors:** Shuai Luo, Qiaoling Zhang, Fan Yang, Jianjun Lu, Zheng Peng, Xiuxin Pu, Juan Zhang, Li Wang

**Affiliations:** ^1^Key Laboratory of Industrial Biotechnology, Ministry of Education, School of Biotechnology, Jiangnan University, Wuxi, China; ^2^Science Center for Future Foods, Jiangnan University, Wuxi, China; ^3^Kweichow Moutai Distillery Co., Ltd., Renhuai, China; ^4^Kweichow Moutai Group, Renhuai, China

**Keywords:** sauce-flavored Daqu, Maillard reaction, melanoidins, enzymatic browning, melanin

## Abstract

Sauce-flavored Daqu exhibits different colors after being stacked and fermented at high temperatures. Heiqu (black Daqu, BQ) with outstanding functions is difficult to obtain because its formation mechanism is unclear. In this study, we compared the metabolites in different types of Daqu using ultra-high-performance liquid chromatography triple quadrupole mass spectrometry to explore the formation process of BQ. We found that 251 differential metabolites were upregulated in BQ. Metabolic pathway analysis showed that “tyrosine metabolism” was enriched, and most metabolites in this pathway were differential metabolites upregulated in BQ. The tyrosine metabolic pathway is related to enzymatic browning and melanin production. In addition, the high-temperature and high-humidity fermentation environment of sauce-flavored Daqu promoted an increase in the melanoidin content *via* a typical Maillard reaction; thus, the melanoidin content in BQ was much higher than that in Huangqu and Baiqu. By strengthening the Maillard reaction precursor substances, amino acids, and reducing sugars, the content of Daqu melanoidin increased significantly after simulated fermentation. Therefore, the enzymatic browning product melanin and Maillard reaction product melanoidin are responsible for BQ formation. This study revealed the difference between BQ and other types of Daqu and provides theoretical guidance for controlling the formation of BQ and improving the quality of liquor.

## Introduction

Chinese liquor is among the most well-known distilled liquors worldwide (Wenchao et al., 2021). It can be approximately divided into 12 types according to different flavor characteristics (Cai et al., 2022), including sauce flavor, strong flavor, and light flavor (Liu et al., 2017). Sauce-flavored liquor is a representative Chinese liquor, similar to Scottish whiskey and French brandy (Wang et al., 2008). Sauce-flavored liquor has a long history of brewing and unique production technology. Daqu provides enzymes and flavor precursors for saccharification, fermentation, and aroma-production during brewing. The fermentation conditions of high temperature and humidity promote the development of Daqu’s unique microbial community structure and abundant enzyme systems (Huang et al., 2017). However, regional differences in temperature and moisture in the fermentation room lead to differences in the appearance of Daqu. Therefore, when a batch of Daqu fermentation is finished and the chamber is dismantled, Daqu is generally divided into three categories based on its color: Baiqu (white Daqu, WQ, 10–15%), Huangqu (yellow Daqu, YQ, 80–85%), and Heiqu (black Daqu, BQ, < 1%) (Gan et al., 2019).

Studies have revealed differences in the microbial community structure and physical and chemical properties of different types of Daqu, but their formation mechanism is unclear (Gan et al., 2019; Deng et al., 2020; Yurong et al., 2021). In actual production, the three types of Daqu are mixed for liquor brewing. However, the process of obtaining different types of Daqu at different time points after unpacking is not well-understood. Uncontrollable formation of Daqu causes large fluctuations in the quality of the liquor between batches (Yurong et al., 2021), and the proportions of the different types of Daqu are random. BQ is considered as one of the main types but its quantity is significantly lower than those of WQ and YQ (Gan et al., 2019). Therefore, analyzing the formation process of BQ is important for exploring the fermentation mechanism of sauce-flavored liquor and improving its quality.

Combined with the significantly darker color of BQ and hot and humid fermentation environment of sauce-flavored Daqu, the products of the Maillard reaction, melanoidins, appear to be responsible for the production of BQ. Substrate amino acids and reducing sugars react under high-temperature conditions to produce melanoidins and browning Daqu (Zheng et al., 2011). Additionally, comparison of the products of the Maillard reaction with metabolites detected in sauce-flavored Daqu revealed numerous common products (Wang et al., 2018), and demonstrated that the Maillard reaction is related to BQ formation. However, the process and products of the Maillard reaction are complex, making studies of Daqu difficult. The Maillard reaction is generally thought to be the main cause of Daqu browning. Enzymes also lead to food browning *via* oxidation. The interaction of phenolic compounds, oxygen, and polyphenol oxidase leads to color reactions that are common in food (Pilizota and Subaric, 1998). Melanin is the final product of enzymatic browning. In recent years, metabolomics methods have been widely used to study Daqu metabolites (Yan et al., 2015; Song et al., 2020; Yang et al., 2021). Here, we evaluated the metabolites of different types of Daqu using metabolomics methods to reveal the influence of the Maillard reaction and enzymatic browning on BQ formation.

This study was conducted to analyze the metabolites of different types of Daqu using non-targeted metabolomics methods and the formation mechanism of BQ from two aspects: enzymatic browning and the Maillard reaction. We used metabolomic analysis methods, such as differential metabolite screening and differential metabolic pathway enrichment, to determine the effect of the enzymatic browning product melanin in promoting the formation of BQ. In addition, by enhancing the substrate amino acids and reducing sugars of the Maillard reaction, we showed that the Maillard reaction can promote the formation of BQ. We determined the formation mechanism of sauce-flavored Daqu and provide a theoretical basis for improving the quality of sauce-flavored liquor.

## Materials and Methods

### Samples and Chemicals

The sauce-flavored high-temperature Daqu samples were provided by Kweichow Moutai Co., Ltd. (Zunyi, China). At the end of Daqu fermentation on day 40, 39 samples were randomly collected from different parts of the Daqu fermentation rooms. After treatment with liquid nitrogen, the samples were stored at −40°C for later use. Among them, BQ 1, BQ 2, and BQ 3 were three parallel samples of BQ; YQ 1, YQ 2, and YQ 3 were three parallel samples of YQ; and WQ 1, WQ 2, and WQ 3 were three parallel samples of WQ. These nine samples were used to evaluate the metabolites of Daqu. BQ I-X, YQ I-X, and WQ I-X were the other 10 samples of BQ, YQ, and WQ, respectively, used to determine the content of melanoidins. The raw materials wheat and mother Daqu for Daqu-making were provided by Kweichow Moutai Co., Ltd.

The chemical reagents used in the experiment were of analytical or chromatographic grade. Methanol, acetonitrile, ammonium acetate, and formic acid were purchased from CNW Technologies (Düsseldorf, Germany). α-Amylase, amyloglucosidase, dispase, and Flavourzyme were purchased from Shanghai Yuanye Biological Technology Co., Ltd. (Shanghai, China). DNS color reagent (3,5-dinitrosalicylic acid) was purchased from Beijing Soleibao Technology Co., Ltd. (Beijing, China). Ninhydrin and reduced ninhydrin were purchased from Sangon Biotech Co., Ltd. (Shanghai, China).

### Daqu Metabolites Extraction

The sample (100 mg) was transferred to an Eppendorf tube. After adding 1 ml of methanol (containing 1 μg/ml internal standard) and 30 s of vortexing, the samples were homogenized at 35 Hz for 4 min and sonicated for 5 min on ice. Homogenization and sonication cycles were repeated 3 times. The samples were incubated for 1 h at −40°C and centrifuged at 12,000 rpm for 15 min at 4°C. The supernatant was stored for liquid chromatography–mass spectrometry (LC/MS) analysis (Cai et al., 2015; Doppler et al., 2016).

### Liquid Chromatography–Mass Spectrometry/Mass Spectrometry Analysis

A ultra-high-performance LC (UHPLC) system with a UPLC HSS T3 column (2.1 × 100 mm, 1.8 μm) coupled to a Q Exactive mass spectrometer (Thermo Fisher Scientific, Waltham, MA, United States) was used for LC-tandem MS (MS/MS) analyses. The mobile phase consisted of 5 mmol/L ammonium acetate and 0.1% formic acid in water in positive mode (A) and acetonitrile (B). The following elution gradient was used: 0–1.0 min, 1% B; 1.0–8.0 min, 1–99% B; 8.0–10.0 min, 99% B; 10.0–10.1 min, 99–1% B; 10.1–12 min, 1% B. The column temperature was maintained at 30°C. The auto-sampler temperature was 4°C, and the injection volume was 2 μl. The QE HFX mass spectrometer was used to acquire MS/MS spectra in information-dependent acquisition mode, which was controlled using acquisition software (Xcalibur, Thermo Fisher Scientific, Waltham, MA, United States). This software was used to acquire the MS/MS spectra using a QE HFX mass spectrometer and continuously acquire the full-scan MS spectrum. The electrospray ionization source conditions were as follows: sheath gas flow rate of 45 Arb; Aux gas flow rate of 15 Arb, capillary temperature of 400°C; full MS resolution of 70,000, MS/MS resolution of 17,500, collision energy of 20/40/60 eV in normalized collision energy mode; and spray voltage of 4.0 kV (positive) or −3.6 kV (negative).

### Raw Data Preprocessing and Annotation

ProteoWizard was used to convert the original data into mzXML format. An in-house program using R and XCMS^1^ was used for peak detection, extraction, alignment, and integration (Dunn et al., 2011). The cutoff value for metabolite annotation was set to 0.3 (Smith et al., 2006) using an in-house MS2 database (BiotreeDB). In basic data analysis, we selected metabolites with an MS2 mass spectrum score of greater than 0.6 for further analysis.

### Principal Component Analysis

The data were automatically modeled and analyzed after logarithmic conversion and centralized formatting using SIMCA software (V15.0.2) (Wiklund et al., 2008).

### Orthogonal Projections to Latent Structures-Discriminant Analysis

SIMCA software was used for log-transformation and UV formatting to reliably screen differential metabolites between groups (Trygg and Wold, 2002). The quality of the model was evaluated using orthogonal projections to latent structures-discriminant analysis (OPLS-DA) modeling and analysis of the first principal component. Seven-fold cross-validation was used for the test. The accuracy of the model was estimated as the R^2^Y (interpretability of the model to categorical variable Y) and Q^2^ (predictability of the model). Finally, a permutation test was conducted to further evaluate the accuracy of the model.

### Differential Metabolites Screening and Volcano Plot Mapping

Compared with univariate statistical analysis methods such as Student’s *t*-test and variance analysis, which focus on independent changes of metabolites, multivariate statistical analysis is helpful to analyze the relationship between metabolites. Considering the results of two types of statistical analysis methods at the same time helps us to observe the data from different perspectives and draw conclusions, and it also helps us avoid false positive or model overfitting caused by using only one type of statistical analysis method (Saccenti et al., 2014); *p*-value < 0.05 and variable importance in the projection (VIP) of the first principal component (PC 1) in the OPLS-DA model of > 1 were used as the screening criteria for differential metabolites and a volcano plot was drawn.

### Differential Metabolic Pathway Analysis for Different Types of Daqu

Complex metabolic reactions in organisms often involve different genes and enzymes, forming a complex metabolic network. Analysis of these metabolic pathways can provide a more comprehensive understanding of the dominant metabolic pathways of BQ compared to those in other types of Daqu. Functional information related to genes and genomes serves as the basis of the Kyoto Encyclopedia of Genes and Genomes (KEGG) Pathway database, and metabolic reactions are used to form metabolic pathways for enzyme-catalyzed reactions (Kanehisa et al., 2016). We performed KEGG metabolic pathway analysis of differential metabolites of BQ and other types of Daqu, obtained all metabolic pathways mapped by the differential metabolites, and analyzed the upregulated metabolic pathways in BQ. In addition, enrichment analysis and topological analysis were performed to comprehensively analyze the metabolic pathways involved in key differential metabolites to identify key differential metabolic pathways (Xia et al., 2015).

### Different Types of Daqu (BQ, YQ, and WQ) Melanoidins Extraction and Quantification

Melanoidins were extracted as described by Bartel et al. (2015) and Ciaramelli et al. (2019) with some modifications. Ten different samples of BQ, YQ, and WQ were collected and crushed. Daqu powder (2 g) was accurately weighed, and 35 ml of 40% ethanol solution was added and immersed in a water bath at 70°C for 3 h. The samples were centrifuged at 6,000 r/min for 10 min to obtain the supernatant, which was evaporated to dryness with a rotary evaporator. The residue was diluted with distilled water in a 50-ml volumetric flask; 40% ethanol was used as a blank control. The absorbance of the samples was measured at 470 nm (Martins and Van Boekel, 2005). The Lambert–Beer law was used to quantify the concentration of melanoidins (Bekedam et al., 2006): Abs = k (L g^–1^ cm^–1^) × concentration (g L^–1^) × optical path length (cm), where C is the melanoidin content (mmol/L), A is the absorbance at 470 nm, k is the molar extinction coefficient of melanoidins of 0.64 L/mmol⋅cm (Martins and van Boekel, 2003), and b is the cuvette thickness (cm).

### Simulated Daqu Production and Fermentation

Because the processes and products of the Maillard reaction are very complicated, the intermediate products cannot be traced without the participation of enzymes. Therefore, our analysis on the Maillard reaction was mainly based on the traceability of materials. Different amylases and proteases were added to hydrolyze the raw materials to provide the starting substrate amino acids and reducing sugars of the Maillard reaction during Daqu production. After fermentation, the melanin content of the amino acids, reducing sugars, and Daqu bricks was determined.

The production of Daqu brick requires 5–10% of wheat flour to pass through a 100-mesh sieve, and 40–60% of fine flour passes through a 20-mesh sieve. The amount of mother Daqu was 7% of the amount of wheat, and the moisture content was 38% of the weight of the Daqu bricks. The size of the experimental Daqu brick was half that of the standard Daqu brick of Moutai, and each Daqu brick weighed 1.25 kg. According to the experiment number in Table 1, amylase and protease were added, and the Daqu brick without enzyme addition was used as a control. After mixing the ingredients, the water, Daqu, and wheat flour formed a uniform sample with no lumps and no dry powder; it could be kneaded into a ball by hand and dispersed when dropped. The formed Daqu bricks had neat edges and corners, no breaks, no dust, tight sides, loose middle, and a turtle-back shape. The prepared Daqu bricks were placed in a constant temperature and humidity incubator for fermentation for 28 days at 30°C and 85% humidity. Samples were collected on days 7, 17, and 28 to determine the contents of reducing sugars and amino acids. According to the standard Daqu brick specification of Moutai Company, the thickest part in the middle was 11–13 cm, the volume of the Daqu bricks was 37 × 28 × 6.5 cm (Zheng et al., 2011), and weight was approximately 10 kg.

**TABLE 1:** Addition of amylase and protease in different experimental numbers.

Experimental numbers	α -Amylase	Amyloglucosidase	Dispase	Flavourzyme
a	−	−	−	−
b	1*(6.7KU)	−	1*(4.8KU)	−
c	3*(20.1KU)	−	3*(14.4KU)	−
d	−	1*(19.6KU)	1*(4.8KU)	−
e	−	3*(58.8KU)	3*(14.4KU)	−
f	1*(6.7KU)	−	−	1*(4.8KU)
g	3*(20.1KU)	−	−	3*(14.4KU)
h	−	1*(19.6KU)	−	1*(4.8KU)
i	−	3*(58.8KU)	−	3*(14.4KU)
j	1*(6.7KU)	1*(19.6KU)	1*(4.8KU)	1*(4.8KU)
k	3*(20.1KU)	3*(58.8KU)	3*(14.4KU)	3*(14.4KU)

1* or 3* indicates that the amount of enzyme added is a multiple of the corresponding enzyme activity of the mother Daqu. α-Amylase: 1U corresponds to the amount of enzyme that liberates 1 μmol maltose per minute at pH 6.9 at 25°C; amyloglucosidase: 1U refers to the amount of enzyme that hydrolyzes soluble starch to produce 1 mg of glucose in 1 ml of enzyme solution at 40°C and pH 4.6, 1 h; Dispase and Flavourzyme: 1U refers to the amount of enzyme used to hydrolyze casein to produce 1 μg of tyrosine under the conditions of 40°C and pH 7.5 in 1 min.

### Determination of Total Amino Acid and Reducing Sugar Content

The ninhydrin color method was used to determine the total amino acid content of the samples (Barbehenn, 1995). To prepare the ninhydrin color reagent, 85 mg of ninhydrin and 15 mg of reduced ninhydrin were dissolved in 10 ml of ethylene glycol methyl ether. To obtain a standard curve, glutamic acid standard solutions were prepared (amino acid: 20, 40, 60, 80, and 100 μg/ml); the same amount of amino acid standard solution, pH 5.4 acetic acid buffer, ninhydrin color reagent, and boiling water were reacted for 15 min. After the reaction solution was diluted with 60% ethanol, the absorbance was measured at 570 nm. The DNS color method was used to determine the reducing sugar content of the samples (Hu et al., 2008). To prepare 0.4, 0.8, 1.2, 1.6, and 2.0 mg/ml glucose standard solutions, equal amounts of DNS solution and glucose standard solution were mixed, boiled in a water bath for 5 min, and cooled; the absorbance of the reaction solution was measured at 540 nm.

To determine the total amino acid and reducing sugar contents of the samples, an appropriate amount of Daqu powder was extracted with an appropriate amount of distilled water. The content of total amino acids and reducing sugars in Daqu was evaluated as described above.

## Results

### Principal Component Analysis for Different Types of Daqu

**FIGURE 1:**
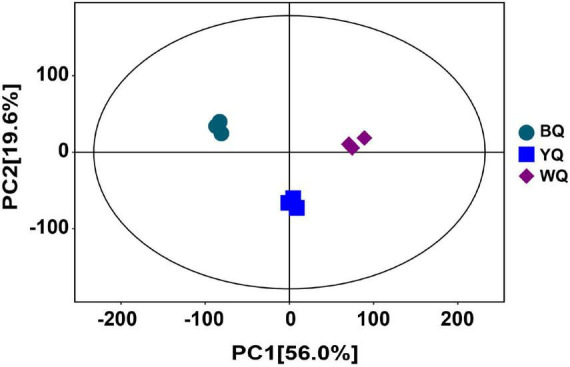
Score scatter plot for PCA model TOTAL.

The base peak chromatograms of the samples are shown in Supplementary Figures 1, 2, where the abscissa represents the retention time of the metabolites, the ordinate represents the relative abundance, and each peak represents the detected metabolite. The metabolite mass spectrum score, metabolite names, and relative quantitative values of the samples are listed in Supplementary Table 1. A total of 1,062 metabolites with different relative abundances were identified in different samples. The principal component analysis (PCA) scores of all samples are shown in Figure 1, along with the first and second principal components, respectively. This result suggests that the metabolites of different types of Daqu were significantly different, but there was no obvious deviation between parallel samples within the group.

### Orthogonal Projections to Latent Structures-Discriminant Analysis for Different Types of Daqu

**FIGURE 2:**
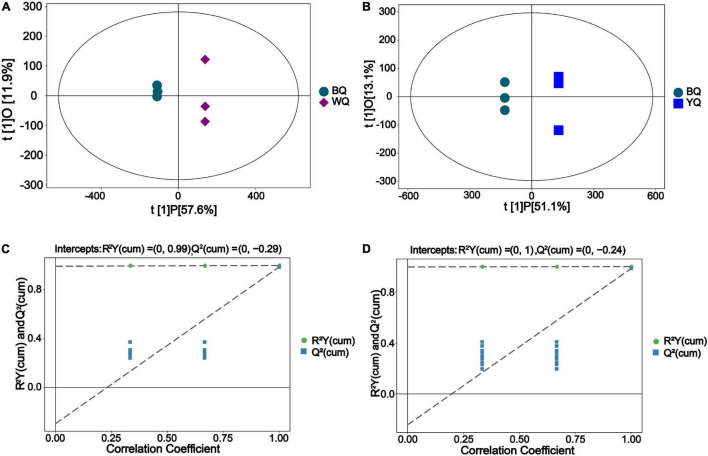
Orthogonal projections to latent structures-discriminant analysis (OPLS-DA) results. **(A)** Score scatter plot of the OPLS-DA model for BQ vs. WQ. **(B)** Score scatter plot of the OPLS-DA model for BQ vs. YQ. The predicted principal component score of PC1 is displayed on the abscissa t[1]P, orthogonal principal component score is displayed on the ordinate t[1]O, and scattered points with different shape and color indicate different samples. **(C)** Permutation test of the OPLS-DA model for BQ vs. WQ. **(D)** Permutation test of the OPLS-DA model for BQ vs. YQ. The abscissa represents the permutation retention of the permutation test (proportion consistent with the Y variable sequence of the original model, and point where the permutation retention is equal to 1 is the R^2^Y and *Q*^2^-value of the original model), and the ordinate represents the regression lines of R^2^Y and Q^2^, respectively.

In OPLS-DA, after orthogonal variables that were not related to categorical variables were screened out, the differences and correlations of metabolites between samples were obtained by analyzing non-orthogonal and orthogonal variables (Wiklund et al., 2008). As shown in Figures 2A,B, the OPLS-DA score results showed that the metabolites of BQ significantly differed from those of WQ or YQ.

In Figures 2C,D, the R^2^Y value in the figure was sufficiently close to 1, indicating that the model fully reflected the true situation of the sample metabolites. In addition, the *Q*^2^-value was similar to the R^2^Y value, indicating that the sample size was sufficient to avoid large errors. Based on these results, differences between samples could be explained by the model. The *Q*^2^-value of the random model permutation test was lower than that of the original model, and the Q^2^ regression line and vertical axis intercept were less than zero. Moreover, the proportion of the Y variable increased as the permutation retention decreased, and the *Q*^2^-value of the random model was consistent with the change in permutation retention. Thus, the original model accurately reflects differences in the samples.

### Differential Metabolites Screening and Volcano Plot Mapping for BQ vs. WQ and YQ

**FIGURE 3:**
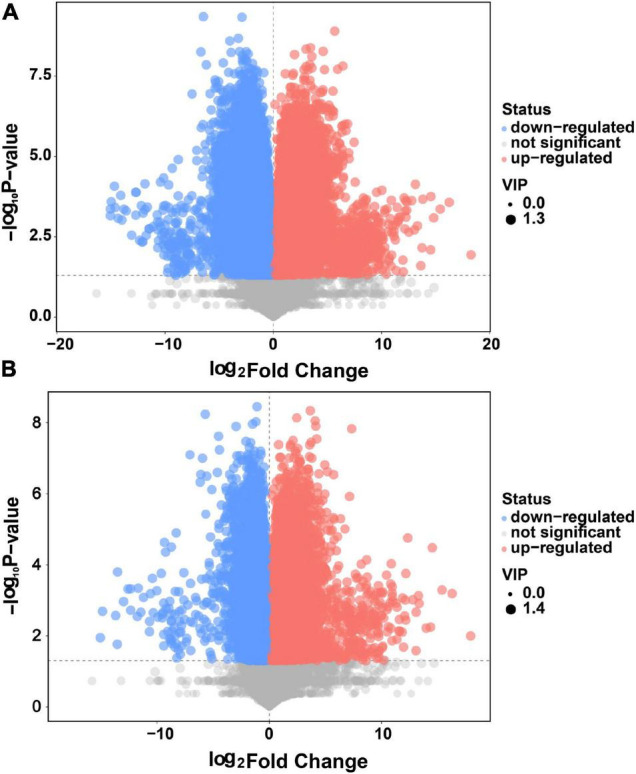
Volcano plot **(A)** of differential metabolites in WQ vs. BQ and **(B)** differential metabolites in YQ vs. BQ. The abscissa is the relative abundance multiple of each metabolite between the two groups (logarithm to base 2), and ordinate refers to the *p*-values of the *t*-test (logarithm to base 10). Different colors of the scatter dots in the figure indicate whether the metabolites were upregulated in BQ. Upregulated metabolites in BQ were red; the metabolites that were downregulated in BQ were blue; gray indicated no significant difference between BQ and other types of Daqu. The variable importance in the projection value of each metabolite is expressed as the area of the scatter point. A larger scatter point indicates a greater variable importance in the projection value of the metabolite.

As shown in Figure 3, 713 differential metabolites were detected between WQ and BQ, and 627 differential metabolites were identified between YQ and BQ (*p* < 0.05, variable importance in projection > 1) (Supplementary Table 2). Among them, 251 common differential metabolites were upregulated in BQ, including 36 organic acids and derivatives; 22 organoheterocyclic compounds; 19 benzenoids; 10 lipids and lipid-like molecules; 8 organic oxygen compounds; 6 nucleosides, nucleotides, and analogs; 5 alkaloids and derivatives; 3 phenylpropanoids and polyketides; 1 organic nitrogen compound; and 1 homogeneous non-metal compound.

We examined the differential metabolites of BQ and other types of Daqu and focused on those that were significantly upregulated in BQ. For example, we detected melanin, the final product of enzymatic browning, and furfural or glyceraldehyde, the typical Maillard reaction intermediate product (Table 2).

**TABLE 2:** Relative abundance of differential metabolites related to enzymatic browning and Maillard reaction upregulation in BQ.

Metabolites	BQ	YQ	WQ
Tyrosine	1.41 × 10^–3^	9.34 × 10^–4^	9.89 × 10^–4^
**Melanin**	9.63 × 10^–5^	7.56 × 10^–5^	5.00 × 10^–5^
Phenylalanine	4.85 × 10^–5^	2.63 × 10^–5^	3.01 × 10^–5^
N-Methyl-2-pyrrolidinone	3.81 × 10^–4^	1.01 × 10^–4^	1.91 × 10^–4^
2-(2-Furanyl)-3-piperidinol	1.77 × 10^–5^	7.45 × 10^–6^	3.36 × 10^–6^
Norfuraneol	9.39 × 10^–5^	5.53 × 10^–5^	4.97 × 10^–5^
2-Benzofurancarboxaldehyde	1.06 × 10^–4^	7.04 × 10^–5^	7.42 × 10^–5^
3-Hydroxypyridine	1.10 × 10^–4^	6.19 × 10^–5^	2.98 × 10^–5^
2-Hydroxypyridine	3.22 × 10^–4^	1.75 × 10^–4^	8.79 × 10^–5^
2-Pyridylacetylglycine	8.28 × 10^–5^	6.83 × 10^–5^	5.19 × 10^–5^
3-Hydroxy-2-Methylpyridine	4.18 × 10^–5^	1.80 × 10^–5^	1.38 × 10^–5^
1-Methyl-6-(1,2,3,4-tetrahydro-6-hydroxy-2-naphthyl)-2(1H)-pyridone	2.98 × 10^–5^	2.02 × 10^–5^	1.43 × 10^–5^
4-Hydroxy-1-(3-pyridinyl)-1-butanone	8.54 × 10^–3^	6.57 × 10^–3^	6.41 × 10^–3^
ETHYL1-BENZYL-3-HYDROXY-2-OXO[5H]PYRROLE-4-CARBOXYLATE	5.07 × 10^–6^	3.57 × 10^–6^	2.45 × 10^–6^
3,4-Dihydro-5-(5-methyl-2-furanyl)-2H-pyrrole	5.58 × 10^–6^	3.68 × 10^–6^	4.04 × 10^–6^
1-(2,3-Dihydro-5-methyl-1H-pyrrolizin-7-yl)ethanone	2.85 × 10^–5^	1.06 × 10^–5^	5.88 × 10^–6^
2,3-Dihydro-5-(3-hydroxypropanoyl)-1H-pyrrolizine	3.29 × 10^–5^	5.45 × 10^–6^	5.66 × 10^–6^
5,6-Dimethylbenzimidazole	2.37 × 10^–5^	1.15 × 10^–5^	1.10 × 10^–5^
Imidazoleacetic acid	1.95 × 10^–5^	7.43 × 10^–6^	8.06 × 10^–6^
2-Amino-1,7,9-trimethylimidazo[4,5-g]quinoxaline	1.67 × 10^–5^	1.01 × 10^–5^	4.88 × 10^–6^
3-Methyl-3H-imidazo[4,5-f]quinoxalin-2-amine	5.86 × 10^–5^	4.08 × 10^–5^	3.84 × 10^–5^
**Glyceraldehyde**	2.65 × 10^–3^	1.75 × 10^–3^	1.96 × 10^–3^
Beta-PHENYL-gamma-Aminobutyric Acid	3.56 × 10^–5^	2.32 × 10^–5^	8.78 × 10^–6^
**Furfural**	1.13 × 10^–4^	6.66 × 10^–5^	4.53 × 10^–5^
3,4-Dihydro-5-(5-methyl-2-furanyl)-2H-pyrrole	5.58 × 10^–6^	3.68 × 10^–6^	4.04 × 10^–6^

### Differential Metabolic Pathway Analysis for BQ vs. WQ and YQ

The regulation of metabolic reactions in organisms is typically complex and mediated by a network of different genes and functional proteins. Their mutual influence and regulation eventually leads to systemic changes in the metabolome (Topfer et al., 2015). Because Daqu fermentation is a mixed bacteria solid-state high-temperature fermentation process, hundreds of microorganisms are involved, and their metabolic pathways cannot be studied individually. *Bacillus* contains a rich raw material hydrolase system, including protease, amylase, cellulase, and glucoamylase, and is considered as one of the most important functional bacterial groups in sauce-flavor Daqu (Yurong et al., 2021). Therefore, we analyzed all differential metabolic pathways of *Bacillus* based on the differential metabolites identified by screening (Supplementary Table 3). Specific enrichment of KEGG metabolic pathways for BQ vs. WQ and YQ is shown in Supplementary Figures 3, 4. Differential metabolites are marked on the KEGG pathway diagram in bright red (indicating upregulation) or bright blue (indicating downregulation). Notably, metabolic pathways related to aromatic amino acid metabolism, such as “phenylalanine metabolism,” “tryptophan metabolism,” and “tyrosine metabolism” in BQ were superior to those in WQ and YQ. These three metabolic pathways include phenylalanine ammonia lyase, peroxidase, and polyphenol oxidase, which can promote enzymatic browning to form BQ.

KEGG annotation analysis identified all pathways involved in the differential metabolites, and further comprehensive analysis was performed using enrichment analysis and topological analysis of the metabolic pathways. We identified the metabolic pathways showing the highest correlation with BQ formation.

**FIGURE 4:**
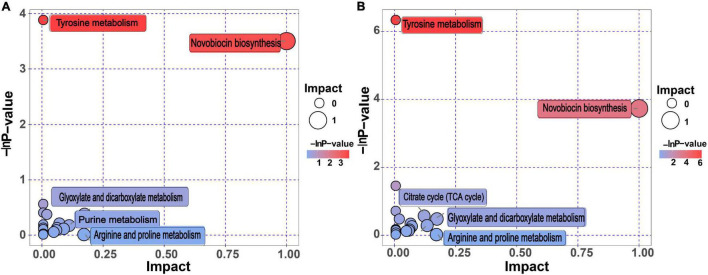
Differential metabolic pathway analysis for BQ vs. WQ and YQ. **(A)** Pathway analysis of WQ vs. BQ. **(B)** Pathway analysis of YQ vs. BQ. The metabolic pathway is represented as bubbles. The size of the bubble represents the impact factor of the metabolic pathway. A larger bubble indicates a larger impact factor of the metabolic pathway in the topological analysis. The color of the bubble indicates the *p*-value (negative natural logarithm, i.e., –ln *p*-value) of the enrichment analysis. A smaller *p*-value with a darker color indicates a more significant difference.

As shown in Figure 4, “tyrosine metabolism” and “novobiocin biosynthesis” were the top two metabolic pathways, both of which had enrichment analysis *p*-values of < 0.05, indicating that these two metabolic pathways had the highest correlation with BQ formation (Figure 4 and Supplementary Table 4). Melanin, the final product of tyrosine metabolism, was identified as a differentially upregulated metabolite of BQ (Table 2), indicating that the tyrosine metabolic pathway is enriched in the Daqu fermentation process. During metabolism, more melanin is produced to promote the formation of BQ.

### Effect of Enzymatic Browning BQ Formation

**FIGURE 5:**
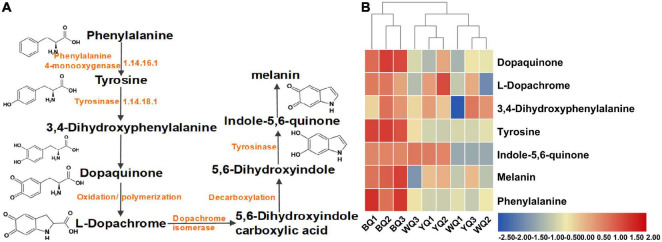
Metabolic pathway of eumelanin biosynthesis. **(A)** Metabolites related to tyrosine metabolism. **(B)** Relative abundance of metabolites related to tyrosine metabolism in metabolomics.

Microbial melanins are typically produced *via* tyrosine metabolism through the DOPA pathway. Tyrosinase, the rate-limiting enzyme of this metabolic pathway, is a polyphenol oxidase that promotes browning. In this pathway, tyrosinase catalyzes the conversion of tyrosine to dopaquinone. Dopaquinone can self-oxidize to form dopachrome, 5,6-dihydroxyindole carboxylic acid, 5,6-dihydroxyindole, indole-5,6-quinone, and eventually form melanin. This type of melanin is known as eumelanin or DOPA-melanin (Tran-Ly et al., 2020; Figure 5A). We analyzed all metabolites detected using LC-MS/MS and found that the relative abundance of most metabolites in this metabolic pathway of BQ was higher than that of YQ and WQ (Figure 5B and Supplementary Table 5). Among them, tyrosine, melanin, and phenylalanine were upregulated in BQ (Table 2), indicating that the tyrosine metabolism pathway was enriched in BQ and produced more melanin through the catalysis of tyrosinase to promote BQ formation. This result is consistent with the conclusion of pathway analysis of differential metabolites, demonstrating that enzymatic browning can promote the formation of BQ.

### Determination of Melanoidins Content of BQ, YQ, and WQ

In addition to enzymatic browning, we analyzed the formation of BQ *via* the Maillard reaction. As shown in Table 3, the melanoidin contents of BQ, YQ, and WQ (10 samples) were BQ > YQ > WQ, indicating that accumulation of melanoidins causes Daqu to exhibit different colors. Daqu, which shows the most vigorous Maillard reaction, eventually forms BQ. In comparison, WQ showed the lowest melanoidin content and corresponded to the least vigorous Maillard reaction. As observed previously, the final product of the Maillard reaction is melanoidins, which causes food to appear as black-brown (Martins et al., 2001). The higher temperature of sauce-flavored Daqu promotes the Maillard reaction to produce melanoidins, resulting in significant browning of Daqu and the formation of BQ.

**TABLE 3:** The melanoidin content of different types of Daq.

Samples	Melanoidin content (mmol/L)	Samples	Melanoidin content (mmol/L)	Samples	Melanoidin content (mmol/L)
WQ I	2.25	YQ I	2.14	BQ I	3.11
WQ II	1.78	YQ II	2.27	BQ II	3.54
WQ III	1.54	YQ III	1.59	BQ III	2.48
WQ IV	1.39	YQ IV	2.77	BQ IV	3.59
WQ V	1.43	YQ V	2.37	BQ V	3.54
WQ VI	1.05	YQ VI	2.55	BQ VI	2.88
WQ VII	0.79	YQ VII	1.97	BQ VII	2.62
WQ VIII	0.73	YQ VIII	2.11	BQ VIII	2.40
WQ IX	1.04	YQ IX	2.51	BQ IX	1.46
WQ X	1.08	YQ X	1.97	BQ X	2.44
Average	1.31 ± 0.44	Average	2.23 ± 0.33	Average	2.81 ± 0.64
*p*-value (WQ vs. BQ)	1.73 × 10^–5^**	*p*-value (YQ vs. BQ)	0.03*	−	−

The p-value of the analysis of variance test that is less than 0.01 is extremely significant**, and less than 0.05 is significant*.

### Analysis of Maillard Reaction Products of Different Kinds of Daqu

**FIGURE 6:**
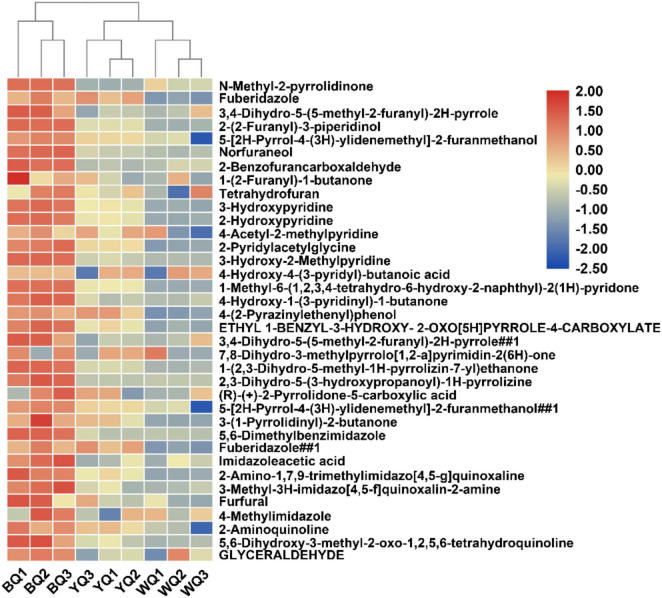
Cluster analysis of relative abundance of Maillard reaction products upregulated in BQ.

Because the concentration of melanoidins in BQ was significantly higher than that in YQ and WQ, we compared the Maillard reaction intermediate products of different types of Daqu, including some common intermediate products of the Maillard reaction, as well as pyrroles, imidazoles, furans, pyridines, pyrazines, oxazoles, and quinolones (Lee and Shibamoto, 2002). Melanoidins are high-molecular-weight polymers produced by the condensation and polymerization of these heterocyclic compounds. A total of 88 Maillard reaction products were analyzed (Supplementary Table 6). Nearly half of the substances were upregulated in BQ vs. in WQ and YQ (Figure 6). Typical Maillard reaction products, such as furfural and glyceraldehyde, were both upregulated in BQ.

### Maillard Reaction Substrate Amino Acids and Reducing Sugars Accelerate BQ Formation

**FIGURE 7:**
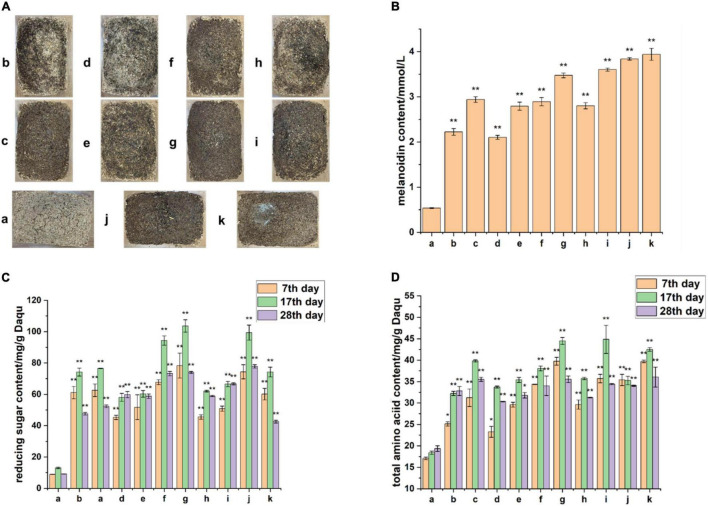
Enhancing Maillard reaction precursor substances to simulate Daqu fermentation. **(A)** Appearance of Daqu at the end of fermentation in the experimental group (b–k) and control group (a). **(B)** Determination of melanoidin content at the end of fermentation. **(C)** Determination of total amino acid content during fermentation. **(D)** Determination of reducing sugar content during fermentation. The **p-value of the analysis of variance test that is less than 0.01 is extremely significant, and *p-value less than 0.05 is significant.

The process and products of the Maillard reaction are complex, and the sources of the products cannot be traced without using enzymes. To further examine the influence of the Maillard reaction on BQ formation, we focused on the substrate supply in the Maillard reaction. By adding amylase and protease during Daqu production, the raw materials of Daqu were hydrolyzed to produce reducing sugars and free amino acids, providing abundant substrates for the reaction. After fermentation, the surface color of Daqu with added amylase and protease was significantly darker than that without enzyme (Figure 7A); the amount of enzyme added was positively correlated with the melanoidin concentration. Monitoring of the fermentation stage also showed that the content of amino acids and reducing sugars in the experimental group with enzymes was much higher than that in the control group without enzymes (Figures 7C,D). This result indicates that the experimental group had sufficient amino acids and reducing sugars as the initial substrate for the Maillard reaction throughout the fermentation stage. This result is consistent with those of Namli, who showed that reducing sugars can promote glycosylation to accelerate the Maillard reaction (Namli et al., 2021), and the amino acid content in BQ was significantly higher than that in YQ and WQ (Deng et al., 2020). Finally, measurement of the melanoidin content confirmed our hypothesis (Figure 7B) that supplying the Maillard reaction precursor amino acids and reducing sugars can accelerate BQ formation.

**FIGURE 8:**
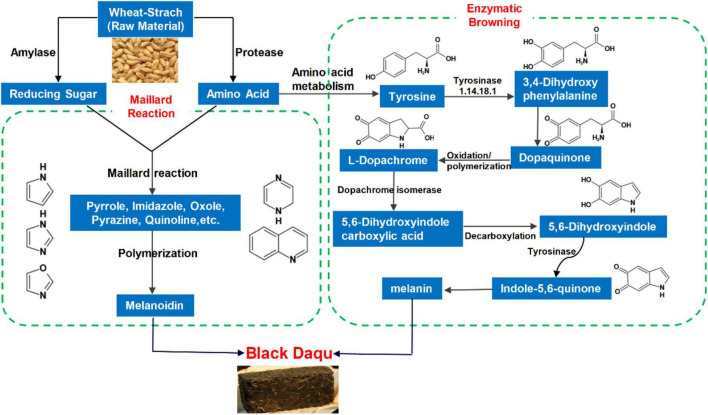
The formation mechanism of BQ.

We examined the formation mechanism of BQ from two perspectives. The raw material wheat was degraded *via* the catalytic activities of amylase and protease to form a large number of amino acids and reducing sugars as precursors for the Maillard reaction. Amino acids and reducing sugars formed intermediates such as pyrazine and furan *via* the Maillard reaction, and finally polymerized to form polymer melanoidins. In addition, in the enzymatic browning process, tyrosinase catalyzes the formation of dopaquinone from tyrosine, and finally forms melanin after oxidative polymerization (Figure 8).

## Discussion

We analyzed the metabolite differences of different types of Daqu samples using non-targeted metabolomics, which showed that sauce-flavored Daqu-BQ formed through enzymatic browning and the Maillard reaction. These findings provide a theoretical basis for identifying different types of Daqu and improving liquor quality.

PCA and OPLS-DA of metabolites in different types of Daqu revealed significant differences in metabolites among BQ, YQ, and WQ. Previous studies of different types of Daqu mainly focused on the microbial community composition and physicochemical properties, whereas different types of Daqu metabolites have not been widely examined. The fungal communities of the three types of Daqu significantly differed, and the biomarkers of WQ, YQ, and BQ were *Thermomyces*, *Thermoascus*, and *Monascus*, respectively (Cai et al., 2021). *Bacillus*, *Staphylococcus*, and *Massilia* can be used as biomarkers for WQ, YQ, and BQ, respectively (Yurong et al., 2021). The differences in the microbial communities of different types of Daqu produce different functional enzyme systems, ultimately leading to differences in metabolic functions.

Differential metabolite analysis showed that among the significantly upregulated differential metabolites in BQ, the abundance of organic acid metabolites was highest, which is consistent with the finding that the acidity of BQ was higher than that of other types of Daqu (Deng et al., 2020). Differential metabolites significantly upregulated in BQ also included a large number of organic heterocycles, including the typical Maillard reaction products furfural and glyceraldehyde. When the substrate is pentosan, furfural is formed by enolization of the Amadori product (Martins et al., 2001). Following the enolization reaction, Amadori products and their dicarbonyl derivatives can simultaneously undergo retro-aldol reactions to produce more reactive C2, C3, C4, and C5 sugar fragments, such as hydroxyacetone derivatives, glyceraldehydes, and diketones (Echavarria et al., 2012). The accumulation of early amylases and proteases exacerbates the Maillard reaction and caramelization, resulting in the formation of more black compounds on the BQ surface (Ban et al., 2015). Our study also supported the findings that adding amylase and protease during Daqu production promoted the Maillard reaction and synthesis of melanoidin, as well as accelerated the formation of BQ (Figure 7). Melanoidins carry a variety of important biological activities. Rufian-Henares and Morales (2007) found that melanoidins isolated from several amino acid-reducing sugar model systems have antioxidant, antimicrobial, and *in vitro* antihypertensive activities. Melanoidins isolated from heated potato fibers by Langner et al. (2011) showed anti-proliferative activity in the culture of glioma cells. The melanoidin concentration of Daqu (particularly BQ) exceeded that of melanoidin produced by the heating model of amino acids and reducing sugar at the same temperature (Martins and Van Boekel, 2005).

The enzymatic browning product, melanin, was also a significantly upregulated differential metabolite in BQ. Metabolic pathway enrichment analysis and topology analysis showed that the synthesis pathway of melanin-tyrosine metabolism was significantly upregulated in BQ. Thus, enzymatic browning was also one factor responsible for the formation of BQ. Melanin has many excellent properties, such as strong free radical scavenging ability (Ma et al., 2018), resistance to ultraviolet radiation (Ye et al., 2019), heat resistance (Li et al., 2019), and antibacterial (Vasanthabharathi et al., 2011) and cell protection (Langfelder et al., 2003), and can be used as antioxidants (Zhao et al., 2022), anti-tumor drugs (Ye et al., 2019), and hair dyes (Battistella et al., 2020). Therefore, studies aimed at screening of melanin-producing bacteria from Daqu and identifying the melanin structure and function are needed.

## Conclusion

In summary, the metabolic components of sauce-flavored Daqu were analyzed using metabolomics combined with simulated Daqu production and fermentation. A total of 251 metabolites were upregulated in BQ. Metabolic pathway analysis showed that “tyrosine metabolism” in BQ was enriched. The end product of this pathway was melanin, and most metabolites in the pathway were upregulated in BQ. In addition, the melanoidin content of the typical Maillard reaction in BQ was much higher than that in YQ and WQ. By increasing the levels of Maillard reaction precursor substances, amino acids, and reducing sugars, we found that the content of Daqu melanoidin increased significantly after simulated fermentation. Therefore, the enzymatic browning product melanin and Maillard reaction product melanoidin are responsible for forming BQ. This study of the formation mechanism of Daqu can provide theoretical support for improving liquor quality and the Daqu production process and provide direction for studies of specific functional metabolites in Daqu.

## Data Availability Statement

The original contributions presented in the study are included in the article/Supplementary Material, further inquiries can be directed to the corresponding authors.

## Author Contributions

SL: conceptualization, data curation, formal analysis, methodology, software, and writing—original draft. QZ: investigation, methodology, resources, validation, and writing—original draft. FY and JL: ethodology, resources, and project administration. ZP: visualization and writing—review and editing. XP: validation, methodology, and resources. JZ and LW: funding acquisition, supervision, and writing—review and editing. All authors have read and agreed to the published version of the manuscript.

## Conflict of Interest

QZ, FY, JL, and XP were employed by the Kweichow Moutai Distillery Co., Ltd. The remaining authors declare that the research was conducted in the absence of any commercial or financial relationships that could be construed as a potential conflict of interest.

## Publisher’s Note

All claims expressed in this article are solely those of the authors and do not necessarily represent those of their affiliated organizations, or those of the publisher, the editors and the reviewers. Any product that may be evaluated in this article, or claim that may be made by its manufacturer, is not guaranteed or endorsed by the publisher.

## Footnotes

1
https://bioconductor.org/packages/release/bioc/html/xcms.html
