# Supplementary material for: Analysis of the Formation of Sauce-Flavored Daqu Using Non-targeted Metabolomics
Source: Front Microbiol. 2022 Mar 24;13:857966. doi: 10.3389/fmicb.2022.857966 (PMC8988067; doi:10.3389/fmicb.2022.857966)
Supplement: Supplementary file 1 [file Data_Sheet_1.ZIP › Support information/Support information.docx]

**Analysis of the Formation of Sauce-flavored Daqu using Non-targeted Metabolomics**

Shuai Luo^1,2,†^, Qiaoling Zhang^3,†^, Fan Yang^3^, Jianjun Lu^3^, Zheng Peng^1,2^, Xiuxin Pu^3^, Juan Zhang^1,2*^ and Li Wang^4*^

^1^Key Laboratory of Industrial Biotechnology, Ministry of Education, School of Biotechnology, Jiangnan University, 1800 Lihu Road, Wuxi 214122, China;

^2^Science Center for Future Foods, Jiangnan University, 1800 Lihu Road, Wuxi 214122, China;

^3^Kweichow Moutai Distillery Co., Ltd., Renhuai, Guizhou, 564501, China

^4^Kweichow Moutai Group, Renhuai, Guizhou, 564501, China

***Corresponding:**

Li Wang

WangliMoutai2021@163.com

Juan Zhang

zhangj@jiangnan.edu.cn

^†^These authors have contributed equally to this work and share first authorship

**Support information**

**Figures**

**
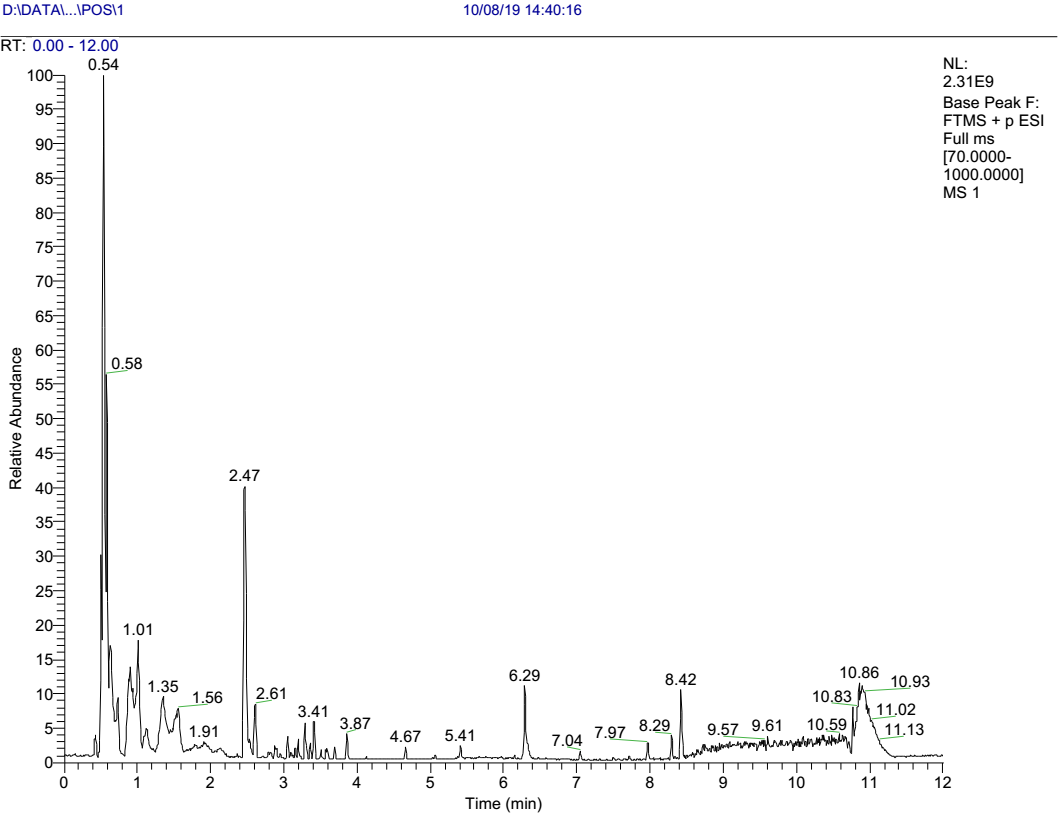
**

**Figure S1** Positive ion mode BPC of the sample detected by UHPLC-QqQ-MS

**
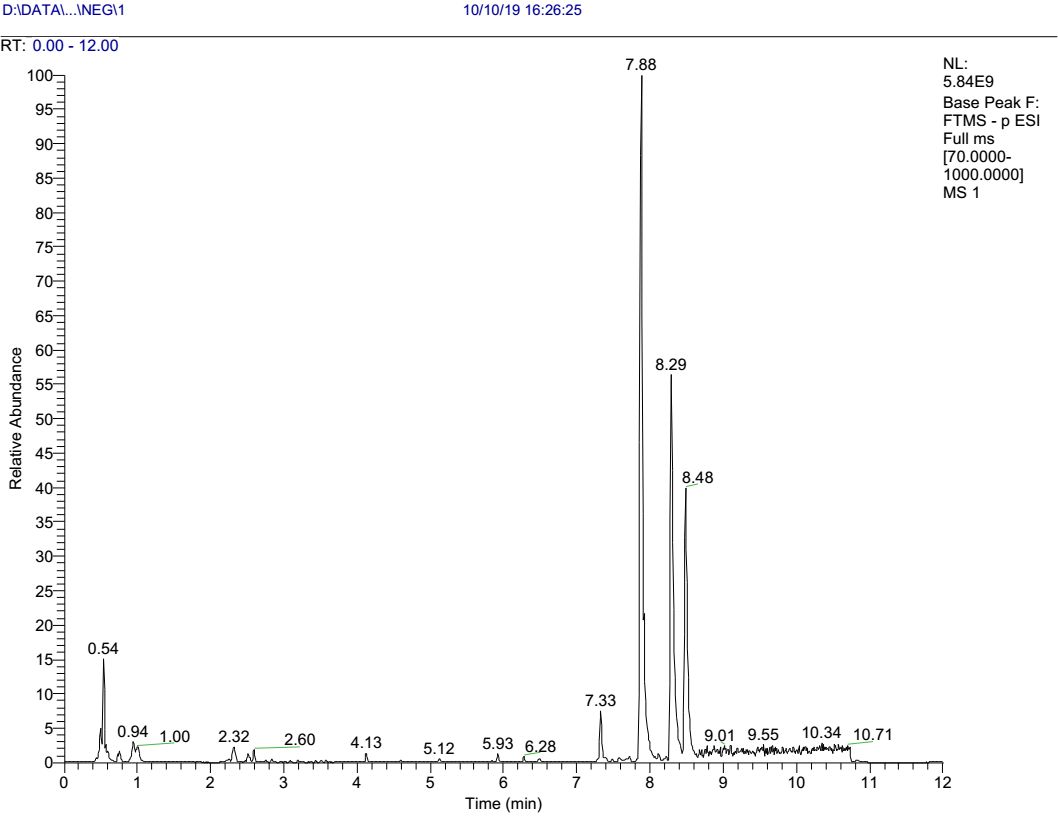
**

**Figure S2** Negative ion mode BPC of the sample detected by UHPLC-QqQ-MS


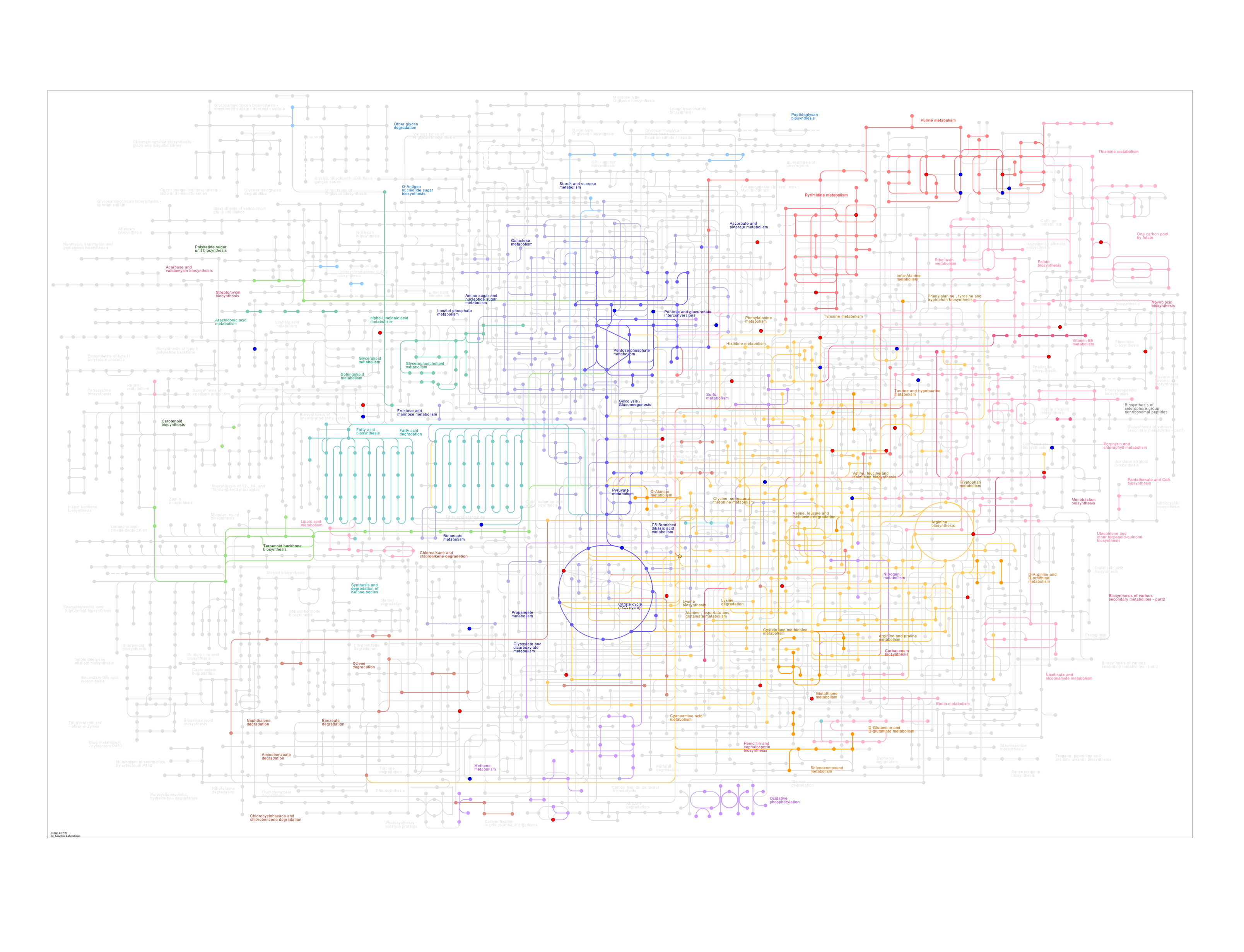


**Figure S3**. Metabolic pathways with different colour dots representing the differential compounds for BQ versus WQ


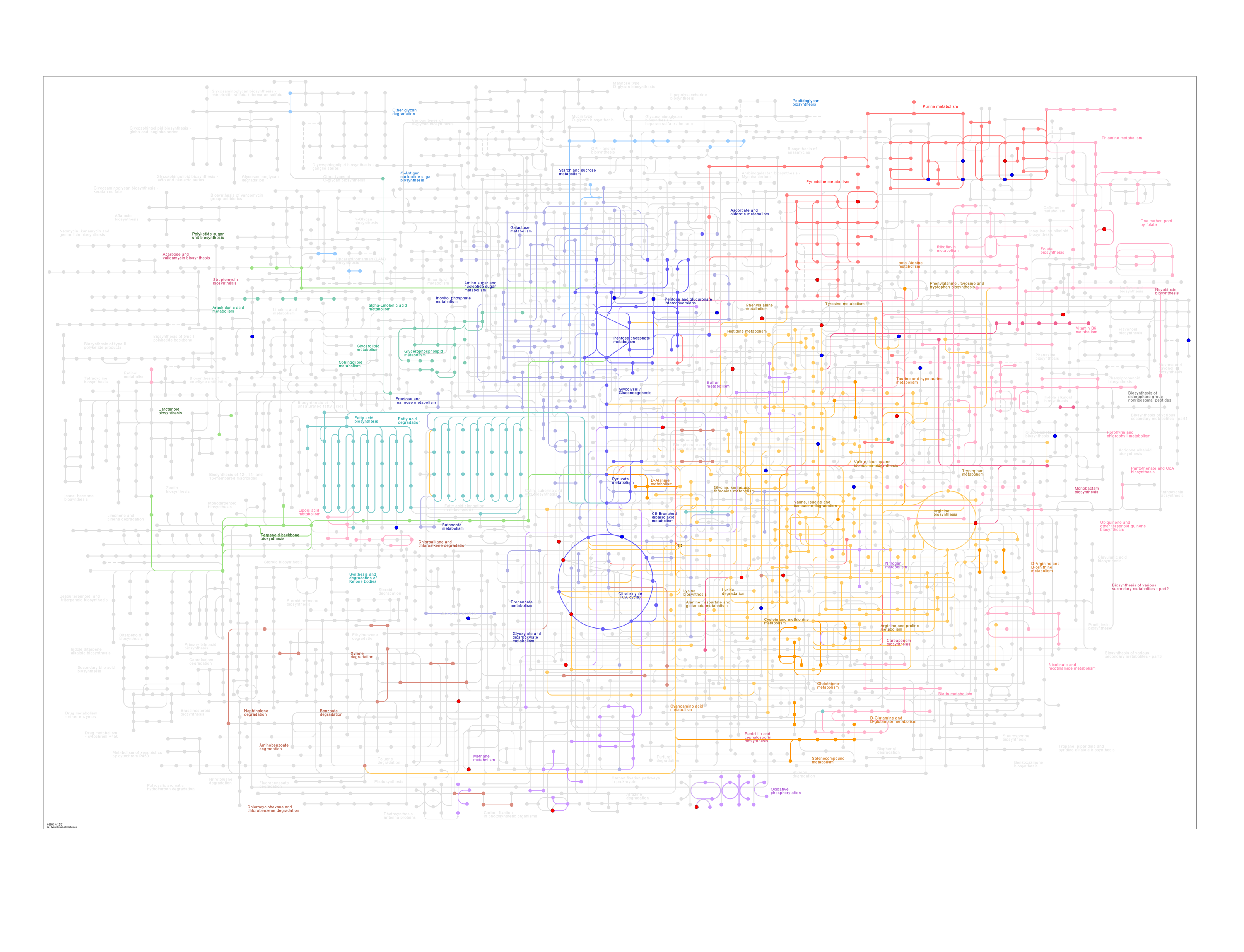


**Figure S4**. Metabolic pathways with different colour dots representing the differential compounds for BQ versus YQ.

**Table S1**. Metabolites mass spectrum score, metabolite names, and samples relative quantitative value.

**Table S2**. Screening of Differential Metabolites of BQ versus WQ and BQ versus YQ.

**Table S3**. All pathways for mapping the differential metabolites of BQ versus WQ and BQ versus YQ.

**Table S4**. Differential metabolic pathway enrichment analysis of BQ versus WQ and BQ versus YQ.

**Table S5**. Relative abundance of metabolites related to tyrosine metabolism pathway in different kinds of Daqu (marked red).

**Table S6**. Relative abundance of Maillard reaction-related products of different kinds of Daqu.
